# Research on Multi-Dimensional Optimal Location Selection of Maintenance Station Based on Big Data of Vehicle Trajectory

**DOI:** 10.3390/e23050495

**Published:** 2021-04-21

**Authors:** Shoujing Zhang, Fujiao Tong, Mengdan Li, Shoufeng Jin, Zhixiong Li

**Affiliations:** 1 Xi’an Key Laboratory of Modern Intelligent Textile Equipment, College of Mechanical and Electrical Engineering, Xi’an Polytechnic University, Xi’an 710048, China; zhangshoujing@xpu.edu.cn (S.Z.); 190211005@stu.xpu.edu.cn (F.T.); 2017022057@stu.xpu.edu.cn (M.L.); 2 Yonsei Frontier Lab, Yonsei University, 50 Yonsei-ro, Seodaemun-gu, Seoul 03722, Korea; zhixiong.li@yonsei.ac.kr; 3 School of Engineering, Ocean University of China, Qingdao 266100, China

**Keywords:** internet of vehicles big data, maintenance station location, k-means algorithm, improved particle swarm algorithm, immune algorithm

## Abstract

In order to rationally lay out the location of automobile maintenance service stations, a method of location selection of maintenance service stations based on vehicle trajectory big data is proposed. Taking the vehicle trajectory data as the demand points, the demand points are divided according to the region by using the idea of zoning, and the location of the second-level maintenance station is selected for each region. The second-level maintenance stations selected in the whole country are set as the demand points of the first-level maintenance stations. Considering the objectives of the two dimensions of cost and service level, the location model of the first-level maintenance stations under two-dimensional programming is established, and the improved particle swarm optimization algorithm and immune algorithm, respectively, are used to solve the problem. In this way, the first-level maintenance stations in each region are obtained. The example verification shows that the location selection results for the maintenance stations using the vehicle trajectory big data are reasonable and closer to the actual needs.

## 1. Introduction

Location selection has an important impact on such aspects as public facilities, maintenance service stations, logistics distribution centers, gas stations, charging stations, and so on. The proper location can not only reduce the cost, but also increase customer satisfaction. The heavy-duty vehicle repair service station is an indispensable piece of infrastructure for vehicle travel. It is responsible for important maintenance and service functions. During the driving of the vehicle, it needs to be maintained, repaired, and replaced from time to time. As the core part of automobile after-sales service, the service station plays a vital role in automobile after-sales service. Therefore, in the fierce modern market environment, the appropriate location of the maintenance station is particularly important. How to determine the location of the maintenance station has become one of the common needs of enterprises and society.

Scholars at home and abroad have done a lot of research on the theory and method of site selection, and achieved corresponding results. Some scholars build quantitative mathematical models through objective function and constraint conditions to study the location problem, and solve it by heuristic algorithm. Dan et al. [1] proposed the application of a stochastic programming model to study the location problem of distribution centers under uncertain demand. Lan et al. [2] studied the location of the distribution center based on uncertain customer demand and the fixed cost of the distribution center built. Some scholars have also proposed multi-level planning site selection, and separately established models for different levels of problems for comprehensive solutions. Wu et al. [3] proposed a two-level programming model combining location optimization and distribution allocation to optimize the location problem, and designed a heuristic solution algorithm combining a genetic algorithm and Frank–Wolfe algorithm. Wei et al. [4] established a multi-objective location model for bus-filling stations with the first objective of minimizing the construction cost of gas stations and the second objective of minimizing the gas-filling costs of all buses. Li et al. [5] used a factor-scoring method to screen the location of logistics supermarkets based on key factors, and then established a two-level planning location model for the initial location of logistics supermarkets.

With the development of big data technology, many scholars apply big data technology to the problem of site selection. Li et al. [6] used big data, such as user payment method and distance, to reasonably select the location of an electricity fee payment point for the rural electric industry business area, which has obvious practical significance. Yang et al. [7] conducted mining and analysis on electric vehicle travel modes and massive movement data to determine the locations of electric vehicle charging piles. This method accurately locates the demand for electric vehicles and increases the accuracy of site selection. Zhang et al. [8] conducted a systematic analysis on the locations of logistics parks using big data, and gave a complete set of park location plans. Wu et al. [9] used big data to select the location of a logistics distribution center, and found out the actual demand location by screening and analyzing customers’ online orders. Wang et al. [10] established an economic model and a location model by analyzing various factors that affect the location of a substation; this was based on a distributed design incorporating various data, such as remote sensing data and environmental factors, and using an analytic hierarchy process combined with big data analysis mode to design and select the sites of substations. In terms of the location of maintenance stations, Ye et al. [11] conducted a nuclear density analysis of travel hotspots, taking maximum coverage as the objective function and considering coordination with the city, to study the location of taxi service stations. Xie et al. [12] and others used the center of gravity method to select alternative repair stations, and then used the analytic hierarchy process and fuzzy evaluation method to select the location of the after-sales service station. This is a common idea and method of site selection.

To sum up, the models and methods of site selection are constantly improving and developing, and the application of emerging technologies also makes site selection more efficient and accurate. However, there is little research on the location of maintenance stations, and especially on the application of big data technology to the location of maintenance stations. Aiming at the problem of heavy-duty vehicle maintenance station location, this paper uses big data technology to collect and organize data and establish a two-dimensional planning location model. First, the trajectory data in the Internet of Vehicles system is collected and processed, and the k-means clustering algorithm is used to analyze and divide the distribution state of vehicle-driving into regions. Then, the location selection of the first-level maintenance stations is considered under the two-level planning based on the location results of the second-level maintenance stations, and the particle swarm algorithm and improved particle swarm algorithm are used to solve the constraint cost minimization model, while the immune algorithm is used to solve the maximum service level model. Finally, an example is given.

## 2. Problem Description and Model Establishment

### 2.1. Problem Description

In the fierce market competition environment of heavy-duty vehicles, improving the after-sales service level is the key to the success of enterprises, and the establishment of a perfect after-sales service system is the top priority. This paper studies the locations of the maintenance stations of Heavy Truck Group Co., Ltd (Baotou, China). The company pays attention to the construction of after-sales service stations, but the original service station can only provide basic repairs and storage functions for some spare parts, which make it difficult to meet the needs of major repair and vehicle maintenance. In addition, due to the different requirements for maintenance services in various regions, the original maintenance station has problems such as insufficient demand, resulting in excessive resource usage, or excessive demand, resulting in insufficient resources and poor service quality. In order to better provide users with more comprehensive after-sales service, the problem of maintenance station location optimization needs to be solved. Therefore, this paper studies the optimization of maintenance station location.

According to the actual needs of heavy-duty vehicles and the requirements of construction cost, the location of maintenance stations is divided into two levels. Table 1 shows the attributes of maintenance stations at all levels. This section constructs the location model of the maintenance station, and the specific problem can be described as follows: on the basis of vehicle trajectory big data, a k-means algorithm and set coverage model are used to select the second-level maintenance stations across the country [13]. The second-level maintenance stations are widely distributed, and are mainly responsible for the daily maintenance activities in a certain area. The speed of response is the standard to evaluate their excellent performance. The second-level maintenance stations are all over the country, which can meet the basic maintenance needs of vehicles. The first-level maintenance station is the maintenance center and distribution center in a certain area, which functions as a hub and can realize the timely deployment of maintenance service resources. Taking the second-level maintenance station as a demand point, combined with the transportation, construction, operation cost, and service level, the location of the first-level maintenance station under multi-dimensional planning is carried out to ensure that users get the best maintenance service and pure spare parts supply in the shortest time.

### 2.2. Establishment of Double-Dimensional Planning Location Model

Most of the traditional multi-objective solutions take cost minimization or revenue maximization as objective functions, which usually contain multiple impact factors, not a single cost factor. In the process of solving the problem, some factors have been quantified, which are usually normalized and ignored, which results in biased results to a certain extent. Therefore, based on this concept, this paper proposes double-dimensional planning, cost minimization, and service level maximization. An improved particle swarm optimization algorithm and immune algorithm are used to solve the model. In the obtained results, a trade-off analysis is carried out, and the final result is obtained. The model is assumed as follows:

1. The first-level maintenance station is transformed on the basis of the second-level maintenance station, so the new first-level maintenance station is selected from the second-level maintenance station that has been selected;
2. The maintenance capacity and inventory capacity of the first-level maintenance station are not limited;
3. The cost of establishing and operating a maintenance station is fixed and known (including its land cost, storage cost, transportation cost, etc.).

#### 2.2.1. Establishment of Cost-Minimization Model

The first-level maintenance station location problem is to select the appropriate place relative to the second-level maintenance station to construct the first-level maintenance station, so as to minimize the sum of the fixed cost of the facility and the associated transportation cost. The model seeks to minimize all kinds of costs, which are mainly divided into two parts:

- The fixed cost required for the construction of the maintenance station, including the expansion’s land cost, construction cost, and management and operation cost;
- Distance cost from demand point to maintenance station and weight cost.

The cost minimization model is shown in Equation (1).

(1) $$MinC=(C_{E}+C_{B}+C_{M})+S_{di}\beta$$

where C is the total cost, $C_{E}$ is land cost, $C_{B}$ is construction cost, $C_{M}$ is the management and operation cost, $S_{di}$ is the distance from the second-level to the first-level maintenance station, and β is the transportation rate from the second-level to the first-level maintenance station, indicating the freight rate per unit of transport weight. I is the first-level maintenance station, expressed as $i_{1},i_{2},\ldots ,i_{n}$; D is the second-level maintenance station (demand point), expressed as $d_{1},d_{2},\ldots ,d_{m}$.

#### 2.2.2. Establishment of Service Level Maximization Model

Service level can be reflected by distance. Under the condition of a certain speed, the closer the distance is, the shorter the time will be. Therefore, it is necessary to calculate the minimum distance between the first-level maintenance station and the second-level maintenance stations in its region, so as to measure the maximum service level. The greater the distance, the lower the level of service, otherwise the opposite. The model of service level maximization is transformed into Equation (2).

(2) $$MaxP_{s}=MinS=Min\sum_{i=1}^{n}\sum_{d=1}^{m}S_{di}y_{di}$$

where $P_{S}$ is the service level, S is the total distance, $S_{di}$ is the distance between second-level maintenance station d (demand point) and first-level maintenance station i (supply point), and $y_{di}$ is the relationship between second-level maintenance station and first-level maintenance station. The constraints are as follows:

(3) $$\sum_{j=1}^{m}y_{di}=1,i\in n$$

(4) $$\sum_{i=1}^{n}r_{i}=k$$

(5) $$y_{di},r_{i}\in \left\{0,1\right\},i\in n,d\in m$$

Among them, k is the number of first-level maintenance stations that need to be selected in the area, and r represents the correlation coefficient. Constraint (3) ensures that each second-level maintenance station (demand point) is only distributed by one first-level maintenance station. Constraint (4) indicates that the total number of selected first-level maintenance stations in the area is k, where $r_{i}$ indicates whether point i is selected as the first-level maintenance station; if it is, it is 1, otherwise it is 0. Constraint (5) means that the two variables are 0–1 variables.

### 2.3. Algorithm Introduction and Design

The advantages and disadvantages of location are reflected in the rationality of the location model algorithm, so it is an NP problem to choose the appropriate model algorithm to solve the location problem. The methods to solve the logistics location problem mainly include precise algorithms and heuristic algorithms. The location of the maintenance station is a large-scale location selection, and an accurate algorithm can only solve the small-scale location optimization problem. In practical problems, when the location scale is large, it is necessary to design a heuristic algorithm to solve the model. Many scholars have also carried out relevant research. For example, the simulated annealing algorithm [14,15], genetic algorithm [16,17], ant colony algorithm [18], particle swarm algorithm [19], immune algorithm [20], and other algorithms are used to solve the location model.

Because there are many demand points and candidate points in this paper, the particle swarm optimization algorithm can speed up the solution of the results by adjusting the number of particles, and the optimization ability is stronger. However, many scholars have improved the particle swarm algorithm in many aspects [21,22,23,24,25], gradually introducing inertia weight, acceleration factor, mixing degree, adaptive factor, and so on. It has been proved by examples that the improved particle swarm algorithm has high efficiency and accuracy. The immune algorithm has a better solution ability in solving multi-value problems. In this paper, the location of the first-level maintenance station is not a single facility, but a group of locations that need to be found, which belongs to a large-scale chain location. Therefore, the improved particle swarm optimization algorithm and immune algorithm are used to solve the double-dimension programming model.

#### 2.3.1. Particle Swarm Optimization Algorithm

Particle swarm optimization (PSO) was proposed by Kennedy J. and Eberhart R. C. in 1995 [26]. It searches for the optimal solution by simulating the foraging behavior of birds. This paper uses the algorithm to optimize the total construction cost of a maintenance station. The equations are as follows:

(6) $$v_{kt}^{k+1}=v_{kt}^{k}+c_{1}r_{1}^{k}(p_{best}-x_{id})best+c_{2}r_{2}^{k}(g_{best}-x_{id})$$

(7) $$x_{kt}^{k+1}=x_{kt}^{k}+v_{kt}^{k+1}$$

(8) $$v_{kt}^{k+1}=\left\{\begin{array}{ll} v_{\max} & v_{kt}^{k+1}>v_{\max}\\ -v_{\max} & v_{kt}^{k+1}<-v_{\max} \end{array}\right.$$

In the above equation, $c_{1}$ and $c_{2}$ are acceleration factor constants; $r_{1}^{k}$, $r_{2}^{k}$ are random numbers in the range of 0–1; v is the particle velocity, $v_{kt}^{k}$ is the velocity of the t-th iteration of the particle (current velocity), $v_{kt}^{k+1}$ is the velocity of the t+1-th iteration; x is the particle position, $x_{kt}^{k}$ is the particle position (current position) of the t-th iteration, $x_{kt}^{k+1}$ is the position of the t+1-th iteration; k is the current number of iterations, $p_{best}$ is the previous best position of a single individual, and $g_{best}$ is the previous best position of the whole population. $\left(p_{best}-x_{id}\right)$ means the particle’s understanding of itself, also known as the “cognition” part, which can guide the particle to its historical best position. The third part $\left(g_{best}-x_{id}\right)$ is called “social knowledge”. Each particle guides all particles in the group to approach the global optimal solution by sharing information. In this paper, on the basis of Equation (6), the weight factor is added to accelerate the global optimal solution. The improved equations are as follows:

(9) $$v_{kt}^{k+1}=wv_{kt}^{k}+c_{1}r_{1}^{k}(p_{best}-x_{id})best+c_{2}r_{2}^{k}(g_{best}-x_{id})$$

(10) $$w=w_{max}-(w_{max}-w_{min})\times \frac{t-1}{T-1}$$

w is the inertia weight of particle change, which is used to indicate the degree of particle keeping the original speed. The inertia weight is set to affect the balance between the local search ability and the global search ability of particles, which mainly represents the impact of the speed of the previous generation on the speed of this generation. The larger the inertia weight w, the greater the influence of the speed of the previous generation on the current, and the particles will move along at the speed of their previous generation to a large extent. T is the maximum number of iterations, and t is the current number of iterations.

The particle swarm optimization method adopts integer coding in the process of locating the first-level maintenance station, and the coding number of particles in the first-level maintenance station corresponds to that of the alternative one. N candidate first-level maintenance station particle A was successively numbered according to 1 to N, and the i-th particle of the first-level maintenance station particle swarm was $A_{i}\left(t\right)=\left\{A_{i1}\left(t\right),A_{i2}\left(t\right),A_{i3}\left(t\right),\ldots A_{i\varphi }\left(t\right),\ldots A_{iJ}\left(t\right)\right\}$ when it evolved into the t-era. If the digit value of $A_{i\varphi }\left(t\right)$ in the particle is n, then this location is the n-th selected first-level maintenance station. If the position is 0, it means that the first-level maintenance station of the position is not selected. The particle B of the second-level maintenance station (demand point) is numbered in sequence from 1 to K, and the i-th particle of the first-level maintenance station particle swarm was $B_{i}\left(t\right)=\left\{B_{i1}\left(t\right),B_{i2}\left(t\right),B_{i3}\left(t\right),\ldots B_{i\varphi }\left(t\right),\ldots B_{ik}\left(t\right)\right\}$ when it evolved into the t-era. If $B_{i\varphi }\left(t\right)$ is m, the demand point will be serviced by the m-th first-level maintenance station selected to be established.

**Figure 1:**
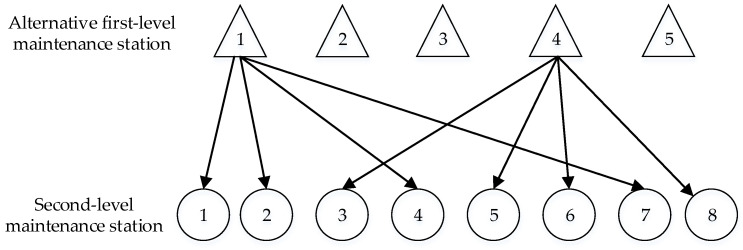
Relational structure diagram of maintenance stations at all levels.

Figure 1 demonstrates the structural relationship between maintenance stations. When two first-level maintenance stations are selected from five alternative first-level maintenance stations for eight second-level maintenance stations, after discretization, particles A and B are $A=\left\{1,0,0,4,0\right\}$ and $B=\left\{1,1,4,1,4,4,1,4\right\}$, respectively. This means that the No. 1 and No. 4 alternative first-level maintenance stations are selected, among which the No.1 first-level maintenance station serves the No. 1, No. 2, No. 4, and No. 7 second-level maintenance stations, and the No.4 first-level maintenance station serves the No. 3, No. 5, No. 6 and No. 8 second-level maintenance stations.

#### 2.3.2. Immune Algorithm

The immune algorithm (IA) was proposed by T. Fukuda and others [27] in 1998. It is similar to the genetic algorithm, which imitates the genetic evolution law of the biological world. The immune algorithm is inspired by the theory of the biological immune system. In this paper, the immune algorithm is used to solve the service level maximization model of the first-level maintenance station.

The initial population of antibodies is generated. The number of selected maintenance stations in the plan is represented by a coded serial number of length p. The coded serial number indicates the serial number of the alternative first-level maintenance station. This case uses the real number encoding method. If the service network consists of 35 second-level maintenance stations, the second-level maintenance stations represented by number $\left\{1,2,\ldots ,35\right\}$ may be selected as the first-level maintenance stations. Then, 2 of the 35 second-level maintenance stations are selected as the first-level maintenance stations. For example, when antibody $\left\{2,8\right\}$ or antibody $\left\{2,14\right\}$ is selected, it means that second-level maintenance stations 2 and 8 or maintenance stations 2 and 14, corresponding to the antibody numbers, will be selected as first-level maintenance stations in the region. This step can ensure that each demand point is met.

The affinity between an antibody and an antigen is used to indicate the recognition of an antigen by an antibody. The function expression is as follows:

(11) $$B_{v}=\frac{1}{\sum_{i=1}^{n}\sum_{d=1}^{m}S_{di}-c\sum_{i=1}^{n}min\left\{\left(\sum_{d=1}^{m}S_{di}\right)-1,0\right\}}$$

In Equation (11), the second term of the denominator is a penalty term, in which the letter c is regarded as a relatively large positive integer, which means that if the distribution distance is too far and exceeds the constraints in the model, it will be punished. $B_{v}$ belongs to the penalty function.

Regarding the affinity between antibodies, the matching degree between antibodies is represented by the method of R-bit continuity. Firstly, the value R is determined, which represents the threshold value for judging the affinity between antibodies. The affinity function $S_{b}$ between antibodies is as follows:

(12) $$S_{b}=\frac{\beta }{l}$$

In Equation (12), β is the same number of digits between antibodies, and l is the length of antibodies. For example, two antibodies with the same length are $\left\{1,2,3,4\right\}$ and $\left\{3,4,5,6\right\}$; after comparison, the two values are the same. In this case, the similarity affinity between antibodies is 0.5.

The antibody concentration is calculated. Antibody concentration is the similar proportion of antibody among all antibodies in the population, which can be expressed as follows:

(13) $$C_{v}=\frac{1}{m}\sum_{i=1}^{n}S_{vi}$$

In Equation (13), $C_{v}$ is the ratio between antibody and antibody group, that is, antibody concentration; M represents the number of antibody species; and $S_{vi}$ represents the similarity between antibody v and antibody i.

Expected reproduction probability is calculated. The expected reproduction probability is also called the incentive degree, which is determined by the affinity between the antibody and the antigen and the antibody concentration. It can be expressed as:

(14) $$E_{v}=\lambda \frac{B_{v}}{\sum_{a=1}^{m}B_{a}}+(1-\lambda )\frac{C_{v}}{\sum_{a=1}^{m}C_{a}}$$

In Equation (14), λ is a constant. From this function, it can be seen that the expected reproduction probability increases with the increase of individual fitness, and decreases with the increase of individual concentration.

This paper uses MATLAB to solve the improved particle swarm optimization and immune algorithm. In order to simplify the understanding of the algorithm solution model, a simple example is given below. In addition, the pseudo code of all algorithms for solving the paper model is shown in Appendix A. An automobile maintenance company plans to select 2 out of 10 second-level maintenance stations to be converted into first-level maintenance stations. Table 2 shows the known attributes of each second-level maintenance station.

Using the above improved particle swarm optimization algorithm and immune algorithm to solve the two dimensions of cost and service level, respectively, the algorithm parameters are consistent with the example verification in the next section, and will not be repeated. The results are summarized as follows:

Table 3 shows the location results of the first-level maintenance station. Scheme 1 is the result of improving the particle swarm to solve the minimum cost, and its service level is calculated by calculating the total distance based on the solved scheme; Scheme 2 is the result of the immune algorithm solving the maximum service level, and the total cost is calculated according to the cost function according to the solved scheme. From the solution results, it can be seen that the total cost and service level of Scheme 1 are better than Scheme 2, so the location scheme is determined as Scheme 1.

## 3. Example Verification

This paper takes the location of the maintenance service station of a heavy-duty truck company as an example, and launches a study on the optimization of the location of maintenance stations nationwide. The vehicle trajectory, historical maintenance records, and current maintenance station location information in the article are all provided by the company. Taking big data such as vehicle trajectory and historical maintenance records, the data is analyzed and summarized. The original data contains a lot of invalid information, so the data needs to be processed. Table 4 shows the information contained in the Internet of Vehicles data. It includes vehicle identification number, vehicle stop point, longitude and latitude coordinates of track, data date, height, mileage, and so on.

**Figure 2:**
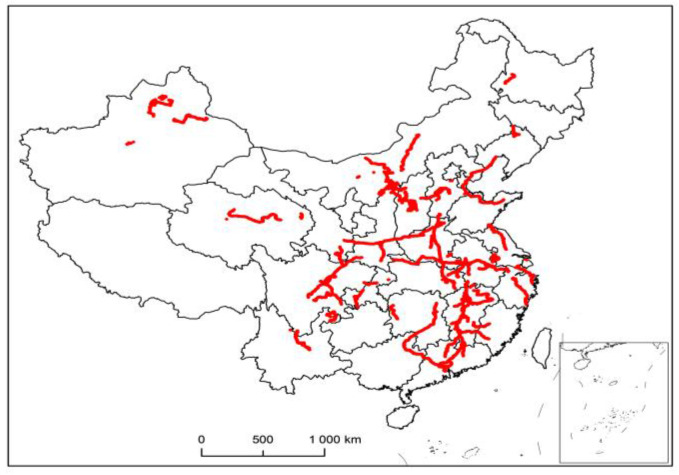
Vehicle trajectory visualization.

When dividing the region, only the latitude and longitude coordinates need to be considered to achieve the clustering effect. Therefore, the data is eliminated, and the effective number of decimal places is retained to obtain the data required by the target. This paper takes driving trajectory, stopping point, and so on, as demand points, that is, the places where vehicles arrive are all demand points. The data of historical records for 10 consecutive days are selected for data cleaning to obtain 76,160 pairs of geographic coordinates. Figure 2 shows the visualization of the vehicle trajectory drawn by ArcGIS.

### 3.1. Region Division of Second-Level Maintenance Stations

In this paper, the big data of vehicle trajectory is processed, and the k-means algorithm and set coverage model are used to select the second-level maintenance stations nationwide. In total, 285 maintenance stations (second-level maintenance stations) are determined nationwide.

**Figure 3:**
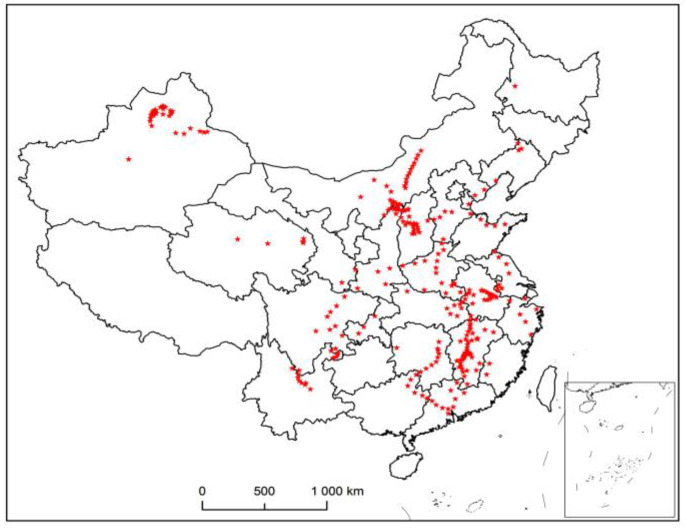
Distribution of second-level maintenance stations.

**Figure 4:**
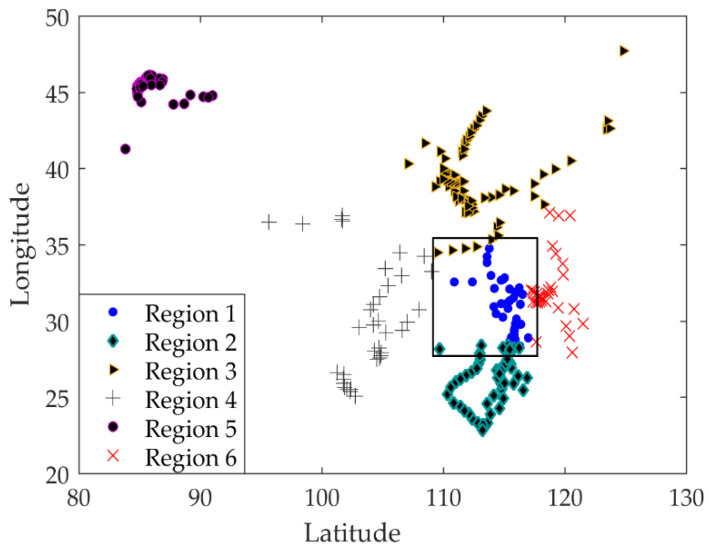
Division of secondary maintenance stations.

Figure 3 shows the distribution map of existing second-level maintenance stations. When combined with Figure 2, it can be seen that the areas where vehicles arrive are relatively scattered, but they are concentrated within the area. In order to determine the location of the first-level maintenance station more reasonably, the second-level maintenance stations in the whole country are divided into regions, and the first-level maintenance station in each region is selected. The track on the left and right side of the upper part of the whole trajectory is dense, and there is no track in the middle part. The northeast corner and northwest corner are recorded as two regions, and the middle part is relatively concentrated and densely distributed. According to the four directions of southeast, northwest, and northwest, they are divided into four regions, and the trajectory is divided into six regions in total. Therefore, the initial clustering data is set as 6. Figure 4 shows the distribution of second-level maintenance stations by using the k-means algorithm.

According to the clustering results, the number of second-level maintenance stations in the six regions is 35, 65, 76, 36, 40, and 33, respectively. The enterprise cost requires that 15 of 285 second-level maintenance stations should be selected as the first-level maintenance stations, and the distribution of each region should be balanced. Each first-level maintenance station needs to meet the demand of 19 second-level maintenance stations, and round off according to a certain linear proportion. Therefore, Table 5 shows that each region contains demand points, and shows the number of maintenance stations at various levels.

### 3.2. Parameter Calculation of Second-Level Maintenance Station

The k-means algorithm divides the second-level maintenance stations nationwide into six regions. Thirty-five second-level maintenance stations in region 1 are selected as examples for verification. Table 6 shows the information of 35 second-level maintenance stations. The actual distance between each second-level maintenance station is calculated by geographic coordinates.

Table 6 shows the attributes of the 35 second-level maintenance stations (demand points) in the first region, including the location, demand, and construction cost of the secondary maintenance stations. Among them, the demand is the weight of spare parts required by each second-level maintenance station, and the fixed cost is the cost of transforming the second-level maintenance station into a first-level maintenance station, including the expansion’s land cost, construction cost, and management and operation cost. The fixed cost is estimated according to the local land rental fee, construction fee, labor cost, and other costs.

### 3.3. Solution of Location Model of First-Level Maintenance Station in Region

**Figure 5:**
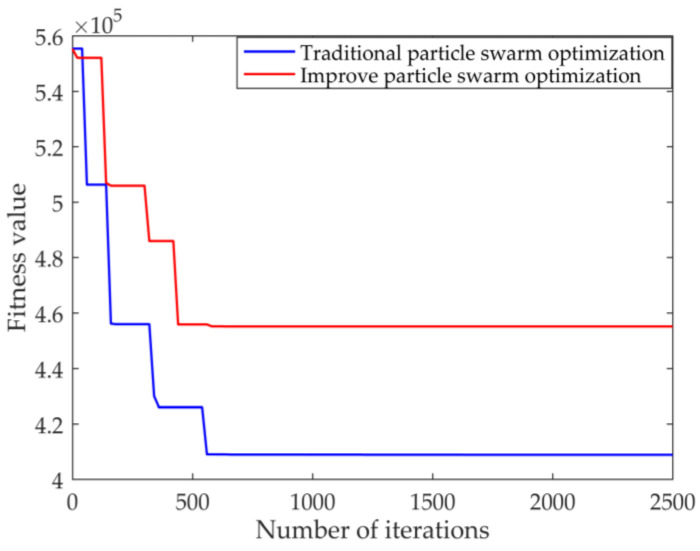
Particle swarm and improved particle swarm algorithm to solve the convergence situation.

Here, the cost minimization model solution is presented. Taking the second-level maintenance station in the first region as the demand point, the above-mentioned attributes are considered; the rate matrix, distance matrix and other combined cost functions are established; and the improved particle swarm algorithm is used to locate the first-level maintenance station in a certain region. The parameters of particle swarm optimization are set as follows: population size *s* = 100, iterations *gen* = 2500, $c_{1}$ = $c_{2}$ = 2, the range of inertia weight of particle change w is 0.4–0.95. Table 7 shows the results of solving the cost function by particle swarm optimization and improved particle swarm optimization, while Figure 5 shows the fitness function.

It can be seen from Table 6 that, using the particle swarm optimization algorithm to solve the cost function, the demand points 14 and 34 are selected as the first-level maintenance stations. The improved particle swarm optimization algorithm selects the demand points 2 and 14 as the first-level maintenance stations, and the cost is lower when the demand points 2 and 14 are selected as the first-level maintenance station. Combining this with Figure 5, it can be concluded that the improved particle swarm optimization algorithm has a faster convergence speed, so the improved particle swarm optimization algorithm has better performance.

**Figure 6:**
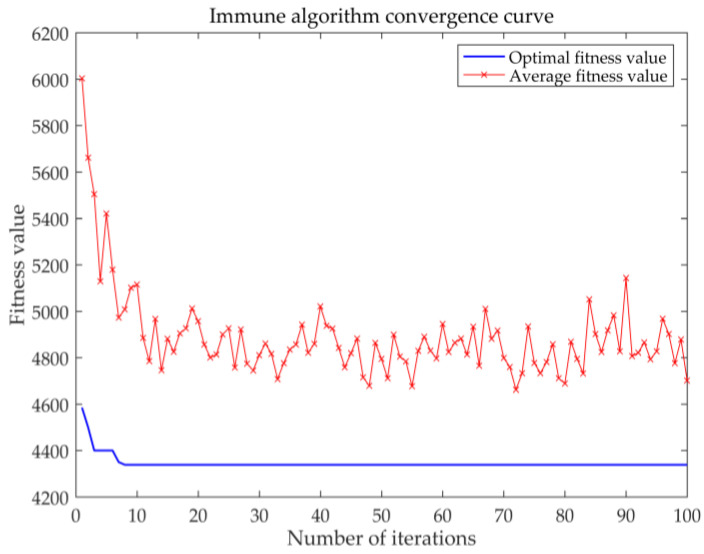
Immune algorithm to solve the convergence situation.

**Figure 7:**
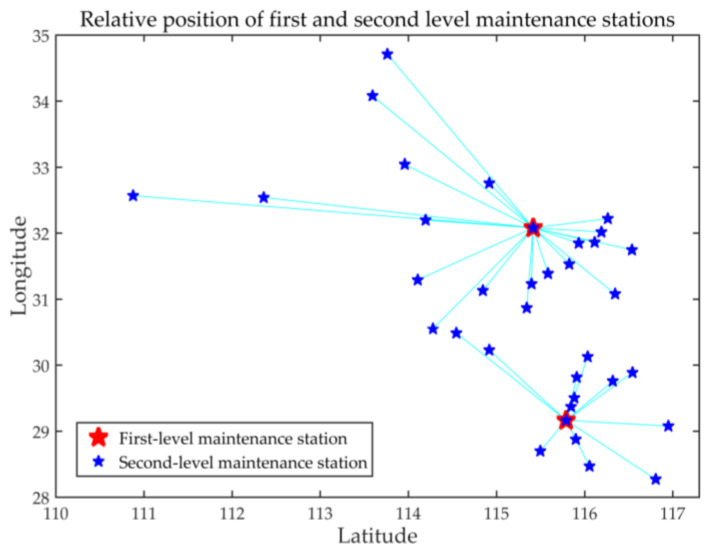
Location distribution of first-level and second-level maintenance stations.

Here, the service level maximization solution is presented. When considering the service level, this paper mainly considers the response of delivery arrival time. Assuming that the rate is constant, the total distance can reflect the quality of service level. The immune algorithm is used to obtain the distribution of the distribution requirements of the second-level maintenance stations in their respective ranges when the total distance is the smallest. The basic parameters of the immune algorithm are set as follows: population size *n* = 50, memory bank capacity *o* = 10, and number of iterations *g* = 100. Crossover probability $p_{c}$ = 0.5, mutation probability $p_{m}$ = 0.4, diversity evaluation parameter $p_{s}$ = 0.95. Table 8 shows the results of the immune algorithm for the service level. Figure 6 shows the optimal distance fitness convergence curve. Figure 7 shows the location distribution relationship between the first-level maintenance station and the second-level maintenance station.

It can be seen from Table 8 that the result of using the immune algorithm to solve the service level (minimum total distance) is that the demand points 27 and 9 are selected as the first-level maintenance stations, and the total distance is 4332 km.

In order to further compare the solution results of the improved particle swarm algorithm and the immune algorithm, the service level (total distance) of the solution solved by the improved particle swarm algorithm and the total cost of the solution solved by the immune algorithm are solved separately. Table 9 shows the comparison solution. Scheme 1 is the result of the immune algorithm solving the maximum service level, and its total cost is calculated according to the solved scheme according to the cost function; Scheme 2 is the result of improving the particle swarm to solve the minimum cost, and its service level is calculated by calculating the total distance based on the solved scheme.

It can be concluded from Table 9 that the result of solving with the minimum total cost as the constraint is that the minimum cost of Scheme 2 is lower than the minimum cost of Scheme 1, and the total cost of Scheme 1 is 1.07 times that of Scheme 2. Taking the service level as the constraint, the result is that the total distance of Scheme 1 is much smaller than that of Scheme 2, and the service level of Scheme 1 is 1.87 times that of Scheme 2. According to the company’s concept of simultaneous development of reducing cost and improving service level, the importance of total distance and service level in the location of maintenance station is set to 1:1, while the actual meaning represented by Scheme 1 is that when the total cost is small, the optimal service level is achieved. Then the location strategy of the first-level maintenance station in the final area is determined as in Scheme 1.

**Figure 8:**
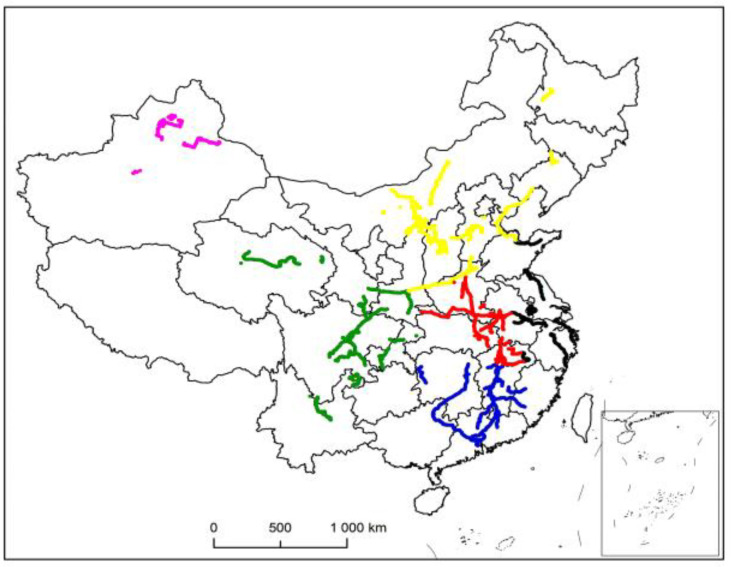
Region division of maintenance station location.

**Figure 9:**
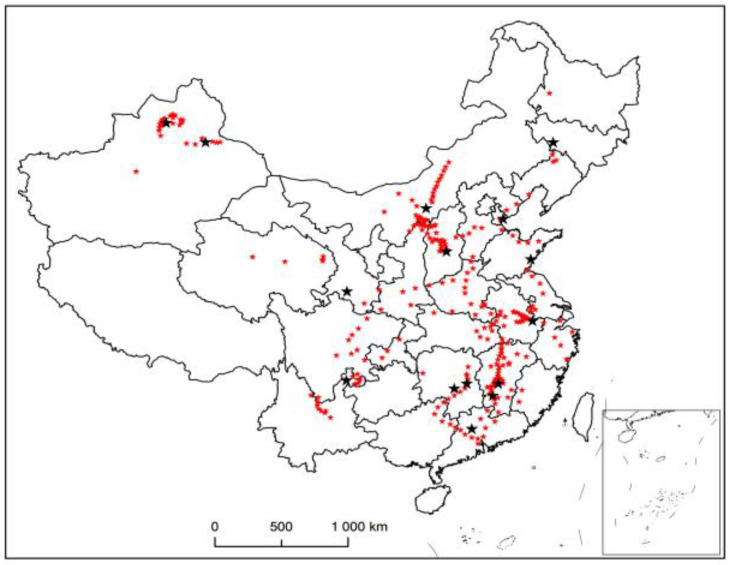
Distribution results of maintenance service stations at all levels.

The other five regions are solved according to the above-mentioned location ideas and algorithms, and the final national first-level maintenance station and second-level maintenance station can be obtained. Figure 8 shows the region division of maintenance station locations. Figure 9 shows the distribution of final location results.

## 4. Conclusions

Based on the big data of the vehicle trajectory, this paper proposes a method for selecting the location of maintenance stations by partition and classification. Taking the big data of vehicle trajectory as the demand point, the maintenance stations are divided into the first- and second-level maintenance stations according to the actual needs of the vehicles, and the responsibilities and functions of maintenance stations at all levels are defined, so as to provide accurate services for vehicles in the region. In addition, the idea of zoning site selection avoids the problem of insufficient site selection results due to uneven demand.

An improved particle swarm algorithm and immune algorithm are used to determine the multi-dimensional location of the first-level maintenance stations. The multi-dimensional planning location model considers all kinds of practical factors, which makes the results more accurate. A two-dimensional planning model is established considering cost minimization and service level maximization, using the improved particle swarm and immune algorithm to determine the first-level maintenance stations. The improved particle swarm can speed up the optimization speed and reduce the cost at the same time, which proves the effectiveness of the algorithm improvement.

However, the above research still has some shortcomings. The paper’s processing of the Internet of Vehicles data is not refined enough, and at the same time, it does not consider updates of the Internet of Vehicles data, that is, it does not consider the dynamic demand. In addition, the paper only considers two aspects of service level and related costs in the location model, and does not consider the model in multivariate situations such as different vehicle types and unavailable service requirements. Therefore, the model proposed in this paper has certain limitations.

Based on the above problems, in the future, this research will study the maintenance demand location problem under dynamic data, and integrate multiple variables into the solution model to enhance the applicability of the location method. At the same time, in terms of algorithm performance, the performance of the algorithm can be improved by performing more refined processing of the initial data of the vehicle trajectory, or adjusting and testing the relevant parameters of the algorithm.

## Figures and Tables

**Table 1:** Attributes of maintenance stations at all levels.

Maintenance Station Level	Main Tasks	Objectives and Requirements	Construction Scale	Service Object
First-level Maintenance Station	Daily maintenance. Emergency maintenance. Spare parts distribution center.	Cost. Quality. Efficiency.	Small quantity. Many service items.	Second-level maintenance station. Vehicle maintenance.
Second-level Maintenance Station	Daily maintenance. Emergency maintenance.	Cost. Quality. Efficiency.	Large quantity. Fewer service items.	Vehicle maintenance.

**Table 2:** Attributes of a company’s second-level maintenance stations.

Demand Point	Coordinate	Fixed Cost (Yuan)	Demand/t
1	1, 2	1100	5
2	3, 3	1600	4
3	5, 9	1400	2
4	8, 2	1400	3
5	3, 6	1500	2
6	6, 9	1300	4
7	7, 3	1800	3
8	4, 8	1200	5
9	1, 6	1100	4
10	2, 6	1400	10

**Table 3:** Attributes of a company’s second-level maintenance station.

Location Scheme	The First-Level Maintenance Station	Service Demand Point	Total Cost/Yuan	Service Level (Total Distance/km)
Scheme 1	1	2 4 5 7	2311	111
	9	3 6 8 10		
Scheme 2	2	1 4 7 9	3122	322
	8	3 5 6 10		

**Table 4:** Data information of Internet of Vehicles (partial information).

Project	Vin	Lng	Lat	Tick_Time	Dir	Height	Mileage	Speed
1	LBZ447DB8HA005216	102.295	25.096	16 October 2018 15:08:34	0	1831	72,224.5	120
2	LBZ447DB4HA005214	102.298	25.119	16 October 2018 09:23:29	0	1816	71,309.3	114.1
3	LBZ447DBXHA005217	102.295	25.088	16 October 2018 09:30:55	0	1806	68,370.4	113.9
4	LBZ447DB5HA005240	109.434	38.993	16 October 2018 10:00:33	180	1280	128,022.1	107
5	LBZ447DBXHA005217	102.295	25.096	16 October 2018 09:31:25	0	1826	68,371.4	106.9
6	LBZ447DB1HA005218	102.300	25.146	16 October 2018 14:47:34	0	1839	66,056.8	104.3
7	LBZ447DB7HA001058	109.434	38.961	16 October 2018 19:19:07	180	1279	135,523	100.5
8	LBZ447DB7HA001061	102.264	24.981	16 October 2018 14:47:34	0	1829	66,037.3	98.2
9	LBZ447DB7HA001058	116.416	31.558	16 October 2018 13:14:46	180	79	83,624.5	95
10	LBZ447DB9HA001059	116.542	31.855	16 October 2018 14:17:34	90	32	150,351	94.5

**Table 5:** Table of clustering results.

Category	Demand Point/Group	Number of Second-Level Maintenance Stations	Number of First-Level Maintenance Stations
Region 1	9220	35	2
Region 2	17,480	65	3
Region 3	20,336	76	4
Region 4	9756	36	2
Region 5	10,585	40	2
Region 6	8783	33	2

**Table 6:** Attribute of secondary maintenance stations.

Demand Point	Geographic Coordinates	Fixed Cost (Ten Thousand Yuan)	Demand/t
1	32.21867, 116.26218	25	28
2	29.16436, 115.79075	20	26
3	31.38921, 115.58497	25	19
4	30.12728, 116.03418	25	14
5	34.71267, 113.76162	45	22
6	32.5403, 112.35854	30	18
7	32.07955, 115.41578	30	13
8	31.84565, 115.93216	25	68
9	32.0177, 116.19089	25	38
10	29.81511, 115.90947	25	10
11	32.19713, 114.19572	30	24
12	32.56858, 110.87686	35	16
13	30.48531, 114.54544	45	9
14	29.50387, 115.88384	20	18
15	28.27018, 116.80671	25	21
16	31.23276, 115.39667	25	34
17	28.87688, 115.90078	35	34
18	30.55213, 114.27848	45	37
19	28.69909, 115.49825	30	18
20	31.13011, 114.84591	30	13
21	31.29302, 114.10686	30	11
22	29.88615, 116.54327	25	16
23	31.07927, 116.34622	30	15
24	33.04207, 113.95872	25	16
25	31.74561, 116.53643	25	25
26	28.4685, 116.05474	35	36
27	30.86745, 115.34308	30	20
28	34.0804, 113.59339	30	67
29	29.37167, 115.84648	25	23
30	29.07695, 116.95104	25	26
31	29.75774, 116.31946	25	42
32	32.75705, 114.91755	30	60
33	31.53074, 115.82617	25	19
34	31.86181, 116.11469	25	40
35	30.22965, 114.91721	30	26

**Table 7:** Summary of cost function solution results.

Solution Algorithm	First-Level Maintenance Station	Include Demand Points	Cost/Yuan
Particle Swarm Optimization	14	2 7 10 11 12 13 14 15 16 17 19 20 21 22 23 24 26 27 29 30 31 33	455,606
	34	1 3 4 5 6 8 9 18 25 28 32 34 35	
Improved Particle Swarm Optimization	2	2 4 5 6 9 10 11 12 13 15 17 18 19 20 21 22 24 26 27 30 33	408,770
	14	1 3 7 8 14 16 23 25 28 29 31 32 34 35	

**Table 8:** Service level solution result.

Solution Algorithm	First-Level Maintenance Station	Include Demand Points	Service Level (Total Distance/km)
Immune Algorithm	27	2 4 10 13 14 15 17 19 22 26 29 30 31 35	4332
	9	1 3 5 6 7 8 9 11 12 16 18 20 21 23 24 25 27 28 32 33 34	

**Table 9:** Summary of site selection schemes.

Location Scheme	The First-Level Maintenance Station	Service Demand Point	Total Cost/Yuan	Service Level (Total Distance/km)
Scheme 1	27	2 4 10 13 14 15 17 19 22 26 29 30 31 35	435,690	4332
	9	1 3 5 6 7 8 9 11 12 16 18 20 21 23 24 25 27 28 32 33 34		
Scheme 2	2	2 4 5 6 9 10 11 12 13 15 17 18 19 20 21 22 24 26 27 30 33	408,770	8105
	14	1 3 7 8 14 16 23 25 28 29 31 32 34 35		

## Appendix A

This appendix is the pseudo code of the entire algorithm for the site selection of the maintenance station in this paper. It is mainly divided into two parts: area division and location model solving. The area division is realized by k-means clustering algorithm, and the two-dimensional location model uses particle swarm algorithm and immune algorithm to solve the minimum cost and maximum service level.

**Pseudo code 1**

The pseudo code of k-means clustering algorithm to achieve area division is described as follows

import vehicle trajectory data points set $D\{x_{1},x_{2},\ldots ,x_{m}\}$

Definition: number of clusters set *k*

**procedure K-means**

 Randomly select *k* samples from *D* as the initial mean vector ${\{u}_{1}{,u}_{2},\ldots {,u}_{k}\}$

 **while true**

 num = 0

 for *i* = 0 to *k*

  $C_{i}$ = [];

 end

for *j* = 1 to *m*

  for *i* = 1 to *k*

  $d_{ji}={\left\|x_{j}-u_{i}\right\|}^{2}$; % Euclidean distance between $x_{j}$ and $u_{i}$

  end

min = $d_{j1}$;

for *i* = 2 to *k*% Find the minimum value in the Euclidean distance

if $d_{ji}$ < min

   min = $d_{ji}$;

   temp = *i*;

  end

 end

lambda = temp;

 C(lambda) = C(lambda)+{$x_{j}$}; % Divide samples into corresponding clusters

 end

 for *i* = 1 to *k* % Update mean vector

 ${u_{i}}^{\prime }$ = Update the mean vector based on the previous cluster;

 if ${u_{i}}^{\prime }$ ~= $u_{i}$

 $u_{i}$ = ${u_{i}}^{\prime }$;

 else

 num+ +;

 end

 end

 if num = = *k*

 break;

  end

 end

 return $C=\{C_{1},C_{2},\ldots ,C_{K}\}$;

**end procedure**

**Pseudo code 2**

The pseudo code of particle swarm to achieve the lowest total cost of repair station location is described as follows:

N is the group size.

**procedure PSO**

   **for** each particle *i*

   Initialize velocity $V_{i}$ and position $X_{i}$ for particle *i*

   Evaluate particle *i* and set $Pbest_{i}$ = $X_{i}$

   **end for**

   *gbest* = min ($Pbest_{i}$)

   **while** not stop

    **for** *i* = l to *N*

    Update the velocity and position of particle *i*

    Evaluate particle *i*

      **if** fit (*i*) < fit ($Best_{i}$)

      $Pbest_{i}$ = $X_{i}$

    **if** fit ($Pbest_{i}$) < fit (*gbest*)

        *gbest* = $Pbest_{i}$:

    **end for**

   **end while**

   print *gbest*

**end procedure**

**Pseudo code 3**

The pseudo code of the immune algorithm to achieve the maximum service level of repair station location is described as follows:

**Procedure IA**

 **Recognize antigen** %Identify the problem to be solved

 **Generate initial antibody population** → Ag %Feasible solution to the initial problem

  **for** each antibody *i*

   fitness(*i*) %Antibody fitness calculation

  **end for**

  **while**(conditions = true)

   print Maxfitness

  **otherwise** calculate antibody concentration and motivation

 Immune treatment{

     Ag1 = Select(Ag) % Immune selection

     Ag2 = Cross(Ag1) % Cross processing

     Ag3 = Mutation(Ag2) % Mutation processing}

  **end while**

  print Maxfitness

**end procedure**
